# Homocysteine Increases Tau Phosphorylation, Truncation and Oligomerization

**DOI:** 10.3390/ijms19030891

**Published:** 2018-03-17

**Authors:** Norimichi Shirafuji, Tadanori Hamano, Shu-Hui Yen, Nicholas M. Kanaan, Hirotaka Yoshida, Kouji Hayashi, Masamichi Ikawa, Osamu Yamamura, Masaru Kuriyama, Yasunari Nakamoto

**Affiliations:** 1 Second Department of Internal Medicine, University of Fukui School of Medicine, Eiheiji-cho, Fukui 910-1193, Japan; sira@u-fukui.ac.jp (N.S.); khayashi@u-fukui.ac.jp (K.H.); iqw@u-fukui.ac.jp (M.I.); kapi@u-fukui.ac.jp (O.Y.); ynakamot@u-fukui.ac.jp (Y.N.); 2 Department of Aging and Dementia (DAD), University of Fukui School of Medicine, Eiheiji-cho, Fukui 910-1193, Japan; 3 Life Science Innovation Center, University of Fukui School of Medicine, Eiheiji-cho, Fukui 910-1193, Japan; 4 Department of Neuroscience, Mayo Clinic Jacksonville, Jacksonville, FL 3224, USA; Yen.Shu-Hui@Mayo.Edu; 5 Department of Translational Science and Molecular Medicine, College of Human Medicine, Michigan State University, Grand Rapids, MI 49503, USA; nkanaan01@gmail.com; 6 National Center for Geriatrics and Gerontology (NCGG), Aichi 474-8511, Japan; hiro@ncgg.go.jp; 7 Department of Neurology, Brain Attack Ota Memorial Hospital, Hiroshima 720-0825, Japan; kuriyama@shouwa.or.jp

**Keywords:** tau, homocysteine, glycogen synthase kinase 3, cyclin dependent kinase 5, caspase 3, protein phosphatase 2A, Alzheimer’s disease, oligomeric tau, vitamin B_6_, vitamin B_12_, folate, S-adenosylmethionine

## Abstract

Increased plasma homocysteinemia is considered a risk factor of dementia, including Alzheimer’s disease (AD) and vascular dementia. However, the reason elevated plasma homocysteinemia increases the risk of dementia remains unknown. A pathological hallmark of AD is neurofibrillary tangles (NFTs) that consist of pathologically phosphorylated tau proteins. The effect of homocysteine (Hcy) on tau aggregation was explored using human neuroblastoma M1C cells that constitutively express human wild-type tau (4R0N) under the control of a tetracycline off system, primary mouse cultured neurons, and by inducing hyperhomocysteinemia in a mouse model of tauopathy (HHCy mice). A wide range of Hcy concentrations (10–1000 µM) increased total tau and phosphorylated tau protein levels. Hcy activated glycogen synthase kinase 3, and cyclin dependent kinase 5, major tau phosphokinases, and inactivated protein phosphatase 2A, a main tau phosphatase. Hcy exhibited cytotoxic effects associated with enhanced activation of caspase. Truncation of tau in the C-terminus, the cleavage site of caspase 3 (i.e., D421, detected by the TauC3 antibody) was also increased. Total tau, phosphorylated tau, as well as C-terminal cleaved tau were increased in the sarkosyl insoluble tau fraction. Hcy also increased the level of tau oligomers, as indicated by the tau oligomer complex 1 (TOC1) antibody that specifically identifies oligomeric tau species, in the tris insoluble, sarkosyl soluble fraction. The levels of TOC1-positive oligomeric tau were increased in brain lysates from HHCy mice, and treating HHCy mice with S-adenosylmethionine, an intermediate of Hcy, reduced the levels of oligomeric tau to control levels. These observations suggest that Hcy increases the levels of phosphorylated tau as well as truncated tau species via caspase 3 activation, and enhanced tau oligomerization and aggregation.

## 1. Introduction

Alzheimer’s disease (AD), progressive supranuclear palsy, corticobasal degeneration, argyrophilic grain disease, Pick’s disease, frontotemporal dementia with parkinsonism linked to chromosome 17, Niemann–Pick disease type C, and chronic traumatic encephalopathy are among a group of disorders collectively known as tauopathies because they are characterized by the accumulation of pathologically modified tau proteins [1,2,3]. A number of factors are considered as important players in causing AD, including aging, oxidative stress, cerebrovascular disorder [4], diabetes [5,6], hypertension [7,8], and hyperlipidemia [9,10]. The mechanisms by which these factors contribute to the formation of abnormal tau inclusions remains unclear.

**Figure 1:**
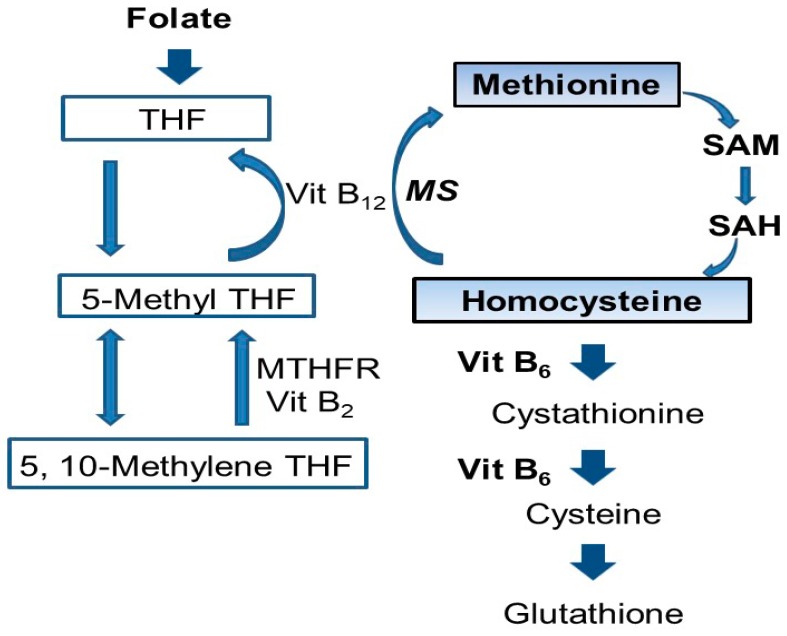
Homocysteine (Hcy) metabolic pathway. Folate and vitamin B_12_ are cofactors in one carbone metabolism, during which they promote the remethylation of homocysteine. Vitamin B_12_ and folate deficiency inhibits metabolism from Hcy to methionine and causes hyperhomocysteinemia (HHCy). Vitamin B_6_ deficiency also inhibits the conversion of Hcy to cystathionine and causes hyperhomocysteinemia. SAM: S-adenosylmethionine; SAH: S-adenosylhomocysteine; Hcy: homocysteine; Vit B_6_: vitamin B_6_, Vit B_12_: vitamin B_12_; MTHFR: 5, 10-methylenetetrahydrofolate reductase; MS: methionine synthase; THF: tetrahydrofolate.

Hyperhomocysteinemia (HHCy) also is considered an important risk factor for dementias, including AD [11] and vascular dementia [12]. Homocysteine (Hcy) is a homologue of cysteine, which is an intermediate in the process of methionine metabolism (Figure 1). 

Homocysteine (Hcy) can lead to DNA breakage, oxidative stress, and apoptosis [13]. Vitamin B_12_ and folate are cofactors in one-carbon metabolism where they promote the remethylation of Hcy, while vitamin B_6_ converts Hcy to cystathionine. Hcy accumulates when there are deficiencies in folate [13], vitamin B_12_ or B_6_ (Figure 1), which may contribute to cognitive dysfunction via HHCy. Hcy increases S-adenosylhomocysteine (SAH) levels, leading to inhibition of S-adenosylmethionine (SAM)-dependent methylation reactions [14] (Figure 1). Hcy can also increase phosphorylation of tau protein via cyclin-dependent kinase 5 (cdk5) activation [15] and inactivation of protein phosphatase 2A (PP2A) [16].

Despite these links between Hcy and tau phosphorylation, it remains unclear whether Hcy facilitates tau oligomerization and truncation. Both phosphorylation and truncation may facilitate tau aggregate formation [17,18]. Indeed, misregulation of kinases and phosphatases has been proposed as a major influencing factor in tauopathies and other disorders with neurodegeneration [19]. Both glycogen synthase kinase 3β (GSK3β) [20] and cdk5 [21] are major tau phosphokinases, while PP2A is a crucial Ser/Thr protein phosphatase that dephosphorylates tau [22,23]. Cleavage of tau protein by caspase at the C-terminus also contributes to tau aggregation and induces cell death [24,25], and recent work demonstrated that upregulation of caspase precedes and results in paired helical filament (PHF) formation [26].

We set out to establish whether HHCy causes tau oligomerization and truncation. We found that Hcy caused tau phosphorylation via activating GSK3β and inactivating PP2A, tau oligomerization, and tau cleavage via caspase 3 activation, and all of these modifications were associated with cell toxicity. HHCy was induced in P301L tau transgenic mice (the Tg4510 line) through a diet deficient in vitamin B_6_, B_12_, and folate. In HHCy mice, an increase of tau oligomerization and activation of caspase 3 were observed. These findings suggest that elevated Hcy contributes to a number of pathological changes in tau protein. 

## 2. Results

### 2.1. Homocysteine Reduces Tau Turnover 

**Figure 2:**
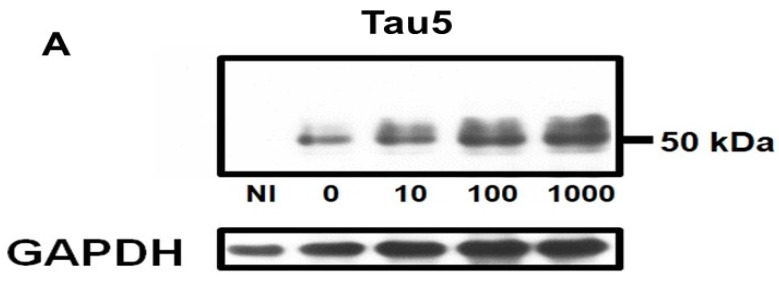

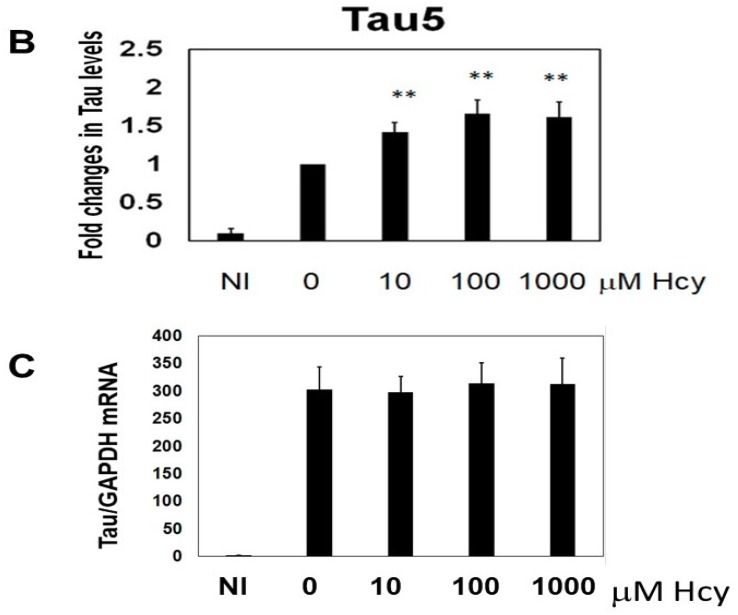
Total tau protein was increased by L-homocysteine (L-Hcy) in M1C cells. M1C cells were subjected to 5 days of tau expression (by reducing tetracycline) and cells were exposed to 10, 100, 1000 µM L-Hcy during the final 24 h of tau induction. The amount of Tau5 positive tau was increased in a dose-dependent manner when cells were treated with L-Hcy. Glyceraldehyde 3-phosphate dehydrogenase (GAPDH) was used as a loading control. Bar: ± SD, ** *p* < 0.01 (**A**,**B**). qPCR showed that mRNA levels of tau did not change with Hcy treatment (**C**). NI: non induced

To test whether Hcy affects tau metabolism, tau expression was induced in M1C cells (by lowering tetracycline from 2 g/mL to 1 ng/mL) for 5 days, and on the 5th day, cells were exposed to L-Hcy (1, 10, 100 or 1000 μM) for 24 h. In clinical settings, the elevation of Hcy is usually mild (>14 M) compared with in our cell-based studies (100 M). The supraphysiological concentration of Hcy was comparable with that in other in vitro studies (50 M, [15]; 150–300 M [27]. Cell lysates were analysed by western blotting using Tau5. Each blot was reprobed with anti- glyceraldehyde-3-phosphate dehydrogenase (GAPDH) antibody to confirm the same loading across the lanes (Figure 2A). Hcy treatment increased total tau protein (Tau5) in a dose-dependent fashion (Figure 2A,B). In cultures treated with 100 μM Hcy, the 45–62 kDa bands detected by Tau5 were increased by 157 ± 45% compared with the control. To establish that the increase in tau was not caused by the increase in tau mRNA expression, mRNA levels were examined. Hcy did not change tau mRNA levels as quantitated by qPCR (Figure 2C).

### 2.2. Homocysteine Increases Phosphorylated Tau 

**Figure 3:**
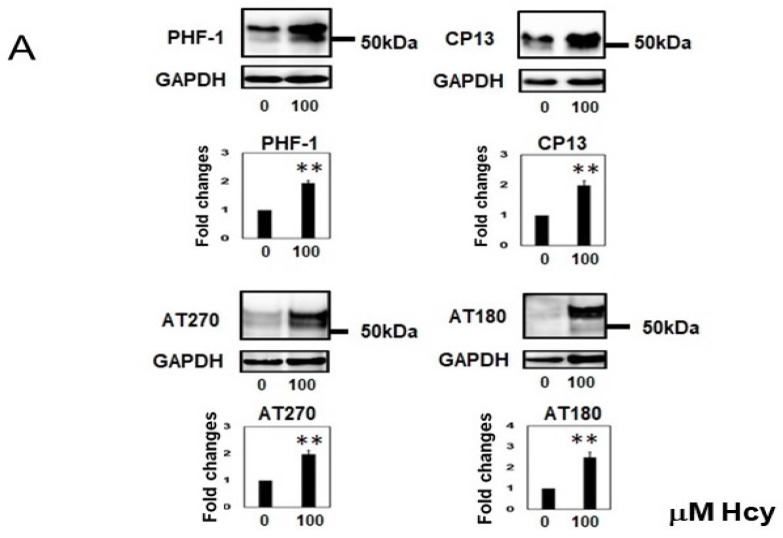

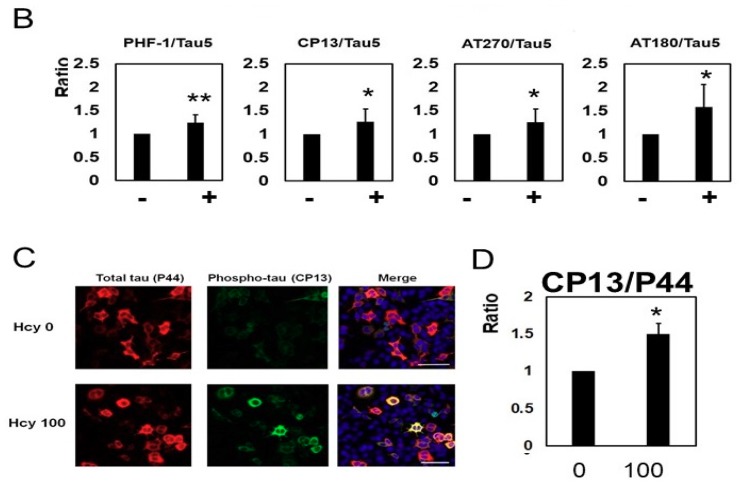
Phosphorylated tau was increased by homocysteine (Hcy) in M1C cells. Hcy increased phosphorylated tau protein. The M1C cells treated with 100 µM Hcy exhibited increases in phosphorylated tau recognized by PHF-1, CP13, AT270, and AT180 antibodies. Bar: ± SD, * *p* < 0.05, ** *p* < 0.01. 0: 0 µM Hcy, 100: 100 µM Hcy (**A**). The phosphorylation ratio was increased by Hcy in M1C cells (µ). −: 0 µM Hcy, +: 100 µM Hcy treated cells. *N* = 4, * *p* < 0.05, ** *p* < 0.01; (**B**). Immunocytochemistry revealed an increase of phosphorylated tau detected by CP13 with Hcy treatment. Hcy 0: 0 µM Hcy, Hcy 100: 100 µM Hcy. Scale: 37.5 µm (**C**). The ratio of CP13-positive cells/P44-positive cells was increased following Hcy treatment. 0: 0 µM Hcy, 100: 100 µM Hcy (**D**).

Phospho-tau levels were analyzed using four phospho-tau antibodies (PHF-1, CP13, AT270, and AT180) and blots were then reprobed with a GAPDH antibody to confirm the same loading. Treating cells with 100 µM L-Hcy increased phospho-tau (52- to 68-kDa bands analyzed) as detected by PHF-1 (192 ± 26%), CP-13 (204 ± 42%), AT270 (195 ± 35%), and AT180 (242 ± 80%) when compared with the control (Figure 3A). The phosphorylation ratio was upregulated by Hcy treatment as shown by PHF-1:Tau5, CP13:Tau5, AT280:Tau5, and AT180:Tau5 ratios (Figure 3B). Immunocytochemical studies using CP13 (Figure 3C) also demonstrated an increase in the ratio of CP13 phosphorylated tau in individual cells expressing tau following Hcy treatment (Figure 3D).

**Figure 4:**
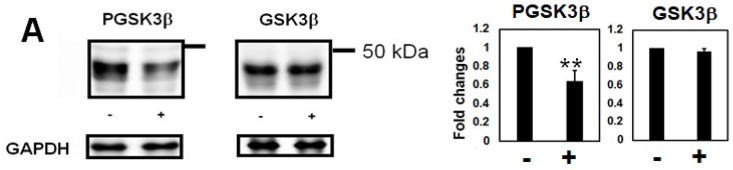

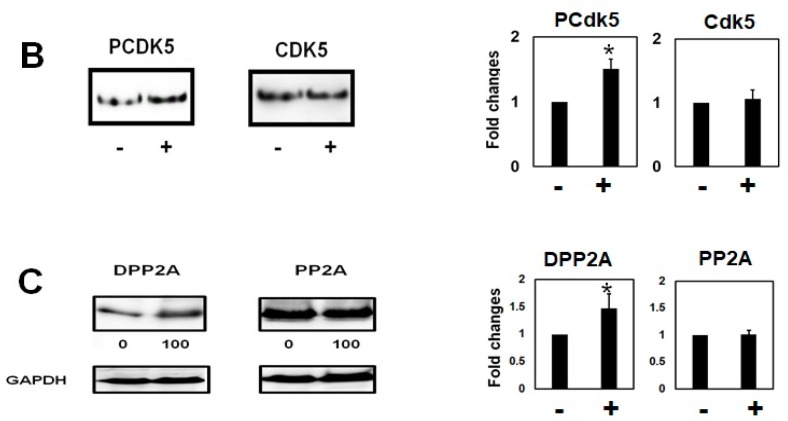
Homocysteine (Hcy) increased GSK3β activity and decreased PP2A activity in M1C cells. (**A**) Hcy activated glycogen synthase kinase 3β (GSK3β). Hcy treatment decreased phospho-S9 GSK3β levels, which suggests active GSK3β was increased. −: 0 µM Hcy, +: 100 µM Hcy treated cells. *N* = 4, ** *p* < 0.01; (**B**) Hcy activated cyclin-dependent kinase 5 (cdk5). Hcy treatment increased phosphorylated cdk5 (Pcdk5). −: 0 µM Hcy, +: 100 µM Hcy treated cells. *N* = 3, * *p* < 0.05; (**C**) Hcy increased the ratio of demethylated protein phosphatase 2A (DPP2A), while the total amount of protein phosphatase 2 (PP2A) did not change, which implies that PP2A (a main tau phosphatase) was inactivated by Hcy. −: 0 µM Hcy, +: 100 µM Hcy treated cells. *N* = 4, * *p* < 0.05, Bar ± SD.

Hcy treatment decreased phosphorylation of Ser9 in GSK3β, which implies that GSK3β activity was increased (Figure 4A). Hcy treatment increased phosphorylation levels of cdk5, which implies cdk5 activity was upregulated (Figure 4B). The catalytic subunit of PP2A was examined by anti-demethylated PP2A (DPP2A, labels inactive PP2A) and anti-total PP2A antibody. DPP2A was increased following Hcy treatment, but total PP2A did not change (Figure 4C), suggesting Hcy inactivated PP2A as previously reported [23].

### 2.3. Homocysteine Treatment Activates Caspase 3 and Increases C-Terminal Truncated Tau

**Figure 5:**
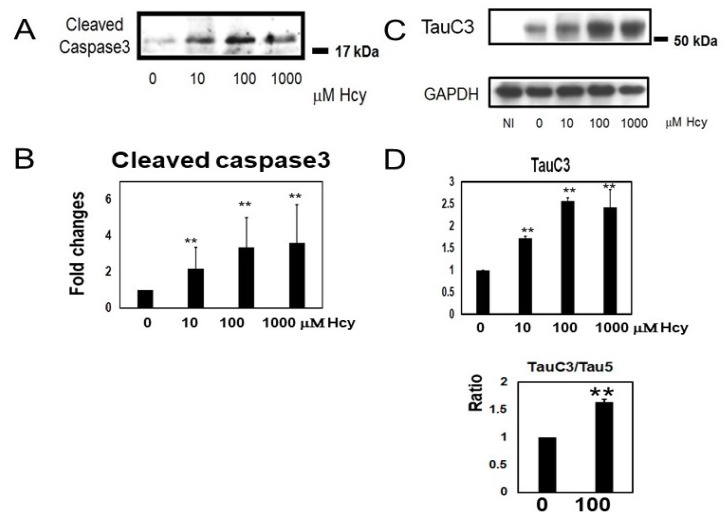

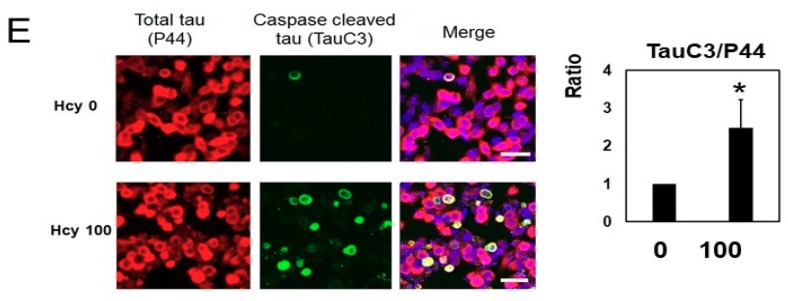
Homocysteine (Hcy) activated caspase 3 and increased caspase-cleaved tau at the C-terminus in M1C cells. Hcy treatment upregulated caspase 3 levels (**A**,**B**). Hcy increases caspase cleavage of tau. TauC3-positive caspase-cleaved tau was increased in a dose-dependent manner when cells were exposed to L-Hcy. The ratio of TauC3 to Tau5 (total tau) was increased by Hcy. Bar: ± SD, * *p* < 0.05, ** *p* < 0.01 (**C**,**D**). Immunocytochemical study demonstrated that homocysteine treatment increased caspase-cleaved tau species. Antibody P44 recognizes the total tau. Treatment of cells with 100 µM Hcy caused a slight increase of Tau5 positive cells. However, TauC3-positive cells markedly increased when cells treated with 100 µM Hcy. The ratio of TauC3 to P44 (total tau) was increased following Hcy treatment. 0: 0 µM Hcy, 100: 100 µM Hcy, Scale: 75 µm (**E**).

A large range of L-Hcy doses (10–1000 µM) activated caspase 3 (Figure 5A,B). The amount of TauC3-positive caspase-cleaved tau was elevated in a dose-dependent fashion when cultures were exposed to L-Hcy (Figure 5C,D). In cultures treated with 100 µM Hcy, levels of total TauC3 positive tau increased (256 ± 8%) compared to vehicle (water) treated control cells and the ratio of TauC3 to Tau5 was increased by Hcy treatment (Figure 5D). Immunocytochemical analysis demonstrated that treatment of cells with 100 µM Hcy slightly increased total tau (as indicated by the P44 tau antibody), but TauC3 positive caspase-cleaved tau was markedly increased with Hcy treatment (Figure 5E). 

### 2.4. Temporal Profile of Phosphorylated Tau and Caspase-Cleaved Tau Following Homocysteine Treatment

**Figure 6:**
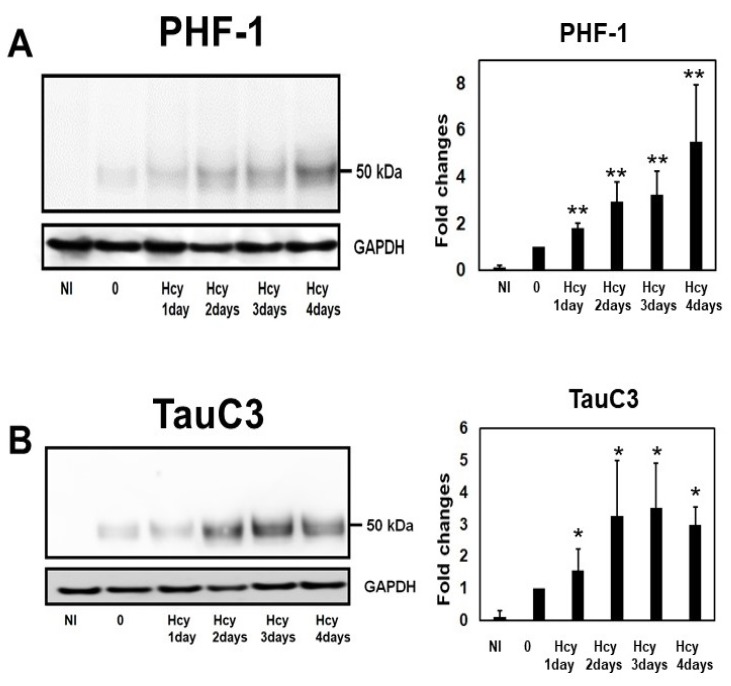
Temporal profile of phosphorylated tau and caspase-cleaved tau following homocysteine (Hcy) treatment. Time courses of phosphorylated tau and caspase-cleaved tau were examined with Hcy (100 µM) treatment for 1 day, 2 days, 3 days, and 4 days. Phosphorylated tau was increased most when the cells were treated for 4 days (**A**). However, the caspase-cleaved tau levels were increased after 2 days of Hcy treatment and remained high through the 4th day (**B**). *N* = 4, NI: non-induced cells, Bar ± SD, * *p* < 0.05, ** *p* < 0.01.

The time courses of phosphorylated tau and caspase-cleaved tau were examined with Hcy (100 µM) treatment for 1 day, 2 days, 3 days, and 4 days. Phosphorylated tau was increased most when the cells were treated for 4 days (Figure 6A). However, the caspase-cleaved tau levels were increased after 2 days of Hcy treatment and remained high through the 4th day (Figure 6B).

### 2.5. Homocysteine Increases Sarkosyl Insoluble Tau and Oligomeric Tau 

**Figure 7:**
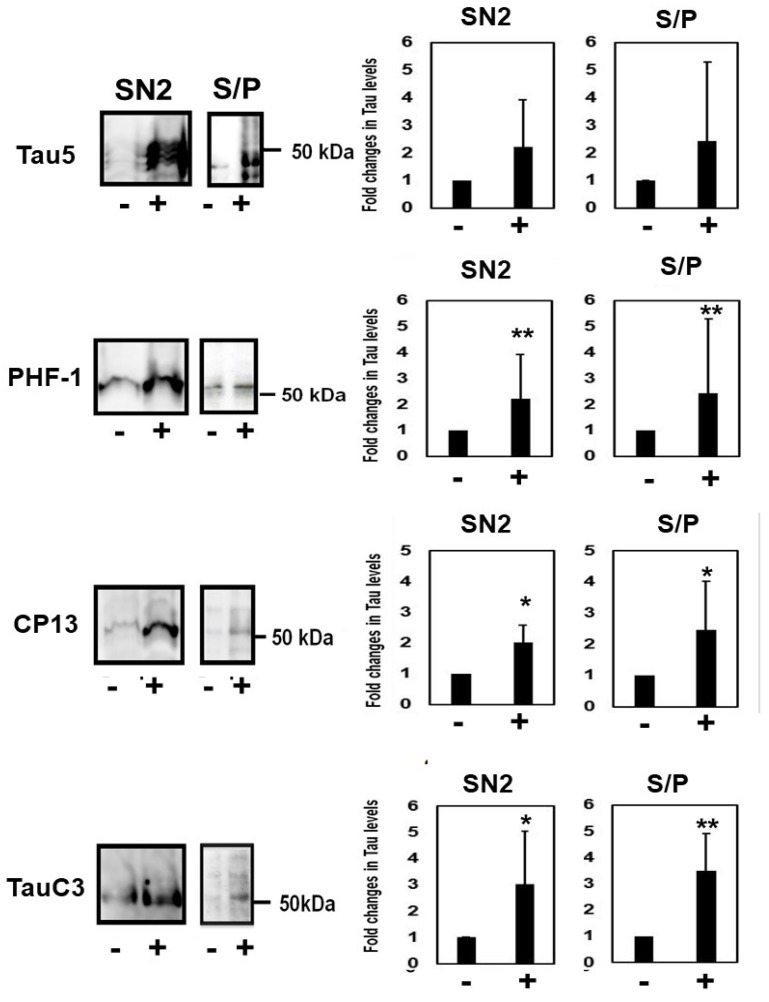
Homocysteine treatment increased sarkosyl-insoluble tau in M1C cells. Lysate from M1C cells induced to express tau for 5 days was treated with 100 µM L-homocysteine (Hcy) on day 4, and then fractionated into SN1 (buffer soluble), SN2 (buffer insoluble and sarkosyl-soluble), and S/P (sarkosyl-insoluble pellet) fractions. Each fraction was analyzed by Western blot analysis using Tau5, PHF-1, CP13, and TauC3 antibodies. Compared with control cells, the increase of tau was observed in all fractions, including the sarkosyl insoluble fraction obtained from cells treated with 100 µM Hcy. −: 0 µM Hcy, +: 100 µM Hcy treated cells. Bar: ± SD, * *p* < 0.05, ** *p* < 0.01.

To examine whether Hcy increases the accumulation of insoluble tau, M1C cells expressing tau for 5 days were treated with 100 µM Hcy on Day 4. Then, cell lysates were fractionated to obtain SN1 (tris soluble), SN2 (tris insoluble sarkosyl-soluble), and S/P (sarkosyl-insoluble) fractions. The fractions were examined by Western blotting using Tau5, CP13, PHF-1, and TauC3 antibodies. Fractionation studies showed that total tau (Tau5), phosphorylated tau (PHF1, CP13), and caspase-cleaved tau (TauC3) in the sarkosyl-insoluble fraction were increased (Figure 7). 

**Figure 8:**
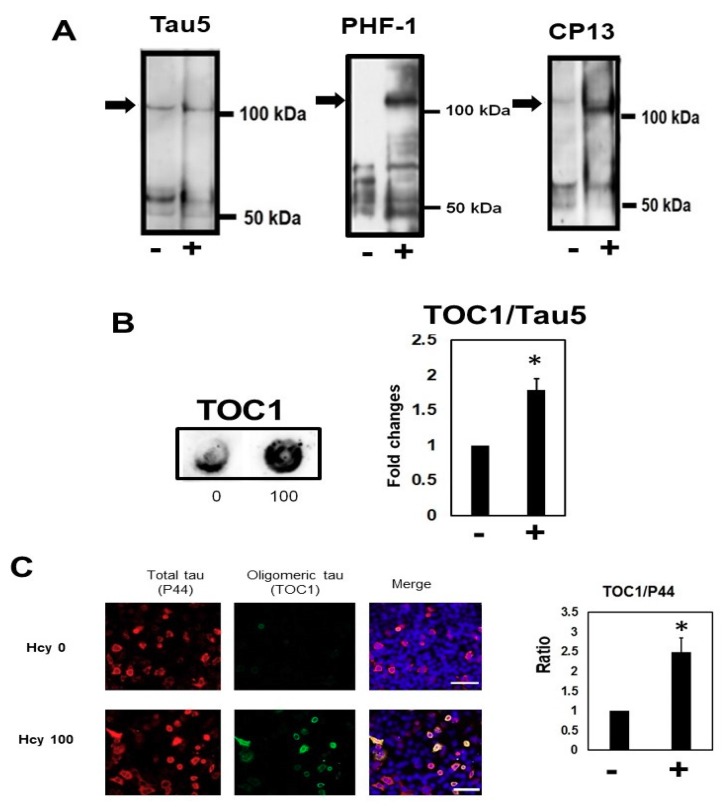
Homocysteine (Hcy) increased oligomeric tau in M1C cells. Hcy increased high molecular weight oligomeric tau in the sarkosyl soluble, tris insoluble (SN2) fraction. Hcy increased total (Tau5) and phosphorylated (PHF-1, and CP13) oligomeric tau (arrow) in the tris insoluble sarkosyl soluble fraction in non-reducing conditions. −: 0 µM Hcy, +: 100 µM Hcy treated cells (**A**). Hcy increased oligomeric tau, detected by tau oligomer complex 1 (TOC1) antibody, in dot blot (**B**) and immunocytochemical studies (**C**). −: 0 µM Hcy, +: 100 µM Hcy treated cells. Bar: ± SD, * *p* < 0.05.

In the tris-insoluble sarkosyl soluble fraction (SN2), Hcy treatment increased high molecular weight tau in non-reducing conditions (Figure 8A). Dot blot analysis (Figure 8B) demonstrated that Hcy increased TOC1-positive oligomeric tau. These immunoblotting data suggest that Hcy increases oligomeric tau species, which we confirmed using TOC1 in immunocytochemical analyses (Figure 8C). 

### 2.6. Homocysteine Induces Cell Death

**Figure 9:**
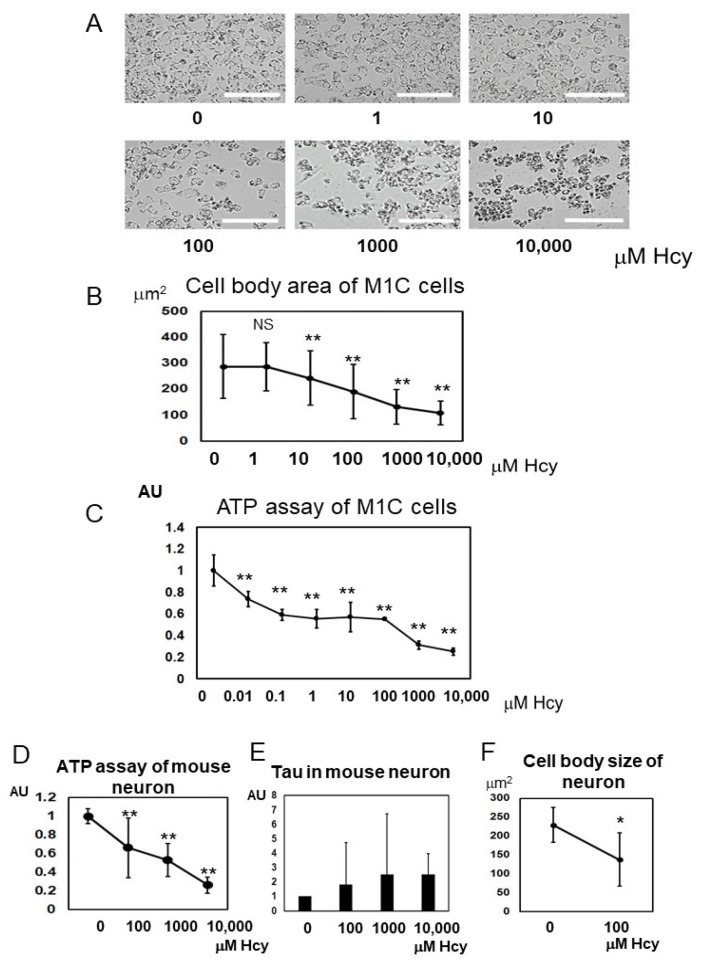
Homocysteine (Hcy) has cytotoxic effects on M1C cells and primary mouse neurons. M1C cells underwent 5 days of tau induction and the cells were exposed to 1, 10, 100, 1000, and 10,000 µM L-Hcy during the final 24 h. Then, M1C cells treated with or without L-Hcy were subjected to morphological studies using a phase contrast microscope to measure the cell body area. Qualitative evaluation of the cultures demonstrates that the cells treated with 100–10,000 µM L-Hcy exhibit shrunken and unhealthy morphologies. Bar 100 µm (**A**). Quantitative evaluation of cell body area showed that 10–10,000 µM L-Hcy treatment caused a significant decline cell size in a dose-dependent fashion. Results are presented as mean ± SD, ** *p* < 0.01 (**B**). The adenosine triphosphate (ATP) assay of M1C cells revealed that Hcy caused a reduction of cell viability. Results are presented as mean ± SD, ** *p* < 0.01 (**C**). ATP assay of primary cultured mouse neuron showed that Hcy has cytotoxic effects on cultured mouse neurons as well. Results are presented as mean ± SD, ** *p* < 0.01 (**D**). Total tau levels in mouse neuron by 10 to 10,000 µM of Hcy treatment were quantitated. Results are presented as mean ± SD (**E**). Quantitative evaluation of cell body area showed that 100 µM L-Hcy treatment caused a significant decline cell size. Results are presented as mean ± SD, * *p* < 0.05 (**F**). NS: not significant.

To study the effects of Hcy on cell morphology and viability, cultured M1C cells on 24-well plates were observed with phase contrast microscopy (Figure 9A). One hundred to 1000 µM L-Hcy induced changes in cell body morphology (i.e., shrinking of the soma). The cell body area was reduced in a dose-dependent manner when M1C cells were treated with 10–10,000 µM Hcy (Figure 9B). Next, we examined whether Hcy caused cell death using an adenosine triphosphate (ATP)-dependent cell viability assay. Low (0.01 µM) to high (10,000 µM) doses of Hcy reduced the number of living cells. The lethal dose, 50% (LD_50_) value was 191.3 µM Hcy for M1C cells (Figure 9C). In non-induced cells (without TetOff induction), the cell body area was reduced by Hcy treatment in a dose-dependent fashion when cells were treated with 10–10,000 µM Hcy. 

Cell viability was decreased by Hcy, in both tau induced cells (Figure 9C), and non-induced cells (Figure S1). Consistent with induction of apoptotic cell death, cleaved caspase 3 was increased by low to high doses of L-Hcy (Figure 5A). The viability of mouse neurons was also measured using the ATP-dependent cell viability assay. One hundred to 10,000 µM L-Hcy induced cell death in mouse primary cultured neurons (Figure 9D).

### 2.7. Homocysteine Induces Endogenous Tau Accumulation

**Figure 10:**
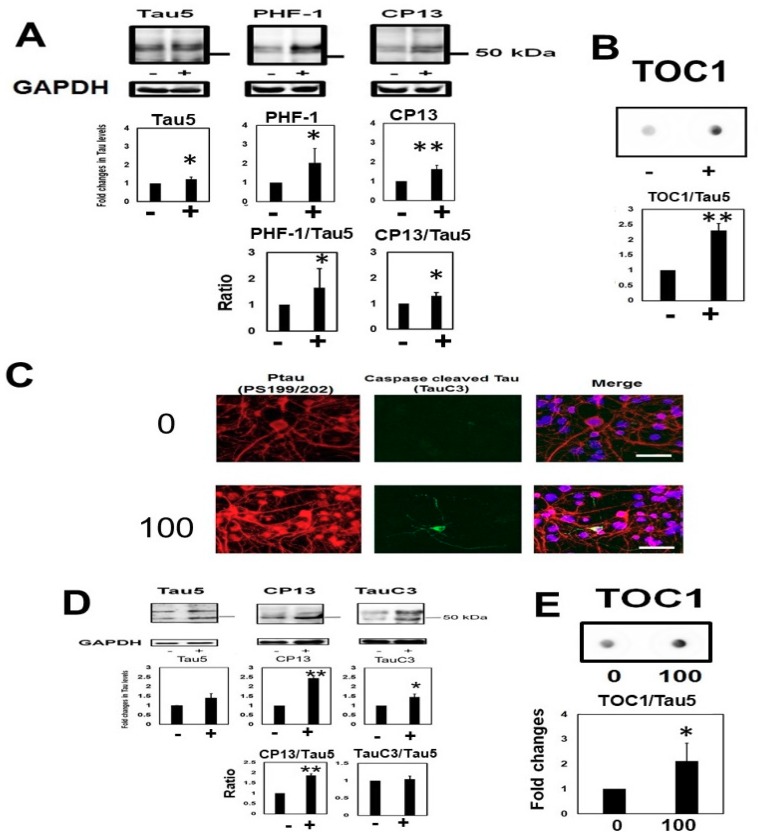
Homocysteine (Hcy) increased endogenous tau in M1Ccells and primary mouse neurons. To examine the effects of Hcy on endogenous tau, non-induced M1C cells (i.e., not expressing exogenous tau) were treated with 100 µM Hcy. Hcy treatment increased total tau (Tau5) and phosphorylated tau (PHF-1, and CP13). −: 0 µM Hcy treated cells, +: 100 µM Hcy treated cells *N* = 4, ± SD, * *p* < 0.05, ** *p* < 0.01 (**A**). The ratio of phosphorylated tau to total tau was increased with Hcy treatment (PHF-1:Tau5, and CP13:Tau5). The data are presented as the relative decrease compared with the DMSO control (*N* = 5), *p* < 0.05 versus control (**A**). Dot blot analysis indicated that oligomeric tau was also significantly increased by Hcy treatment (**B**). Primary neuronal cultures of mice were also treated with 100 µM Hcy. Hcy-treated neurons exhibited an accumulation of phosphorylated tau protein (PS199/202) and C terminal truncated tau species (TauC3). 0: 0 µM Hcy, 100: 100 µM Hcy treated cells. Bar: 35 µm (**C**). Western blot analysis showed that Hcy treatment increased total tau (Tau5), phosphorylated tau (CP13), and C-terminally truncated tau in primary neuronal culture. −: 0 µM Hcy treated cells, +: 100 µM Hcy treated cells *N* = 3, ± SD, * *p* < 0.05, ** *p* < 0.01. Hcy increased the ratio of phosphorylated tau to total tau (PHF-1:Tau5, CP13:Tau5). The data are presented as the relative decrease compared with the dimethyl sulfoxide (DMSO) control (*N* = 3), *p* < 0.05 versus control (**D**). Dot blot analysis showed that Hcy (100 µM) increased TOC1 positive oligomeric tau. *N* = 4, * *p* < 0.05 versus control. Bar: ± SD (**E**).

To evaluate the effects of Hcy on endogenous tau, non-induced M1C cells (i.e., maintained in 2 µg/mL Tet) were exposed to 100 µM Hcy. Hcy increased both total tau (Tau5), and phosphorylated tau (PHF-1, and CP13) (Figure 10A). The ratio of phosphorylated tau (PHF-1, and CP13) also increased with Hcy treatment. Dot blot analysis indicated that oligomeric tau was also significantly increased by Hcy treatment (Figure 10B). Primary neuronal cultures of mice were exposed to 100 µM Hcy. Hcy-treated neurons exhibited accumulation of phosphorylated tau (CP13), as well as C-terminal truncated tau protein, especially in the cytosol (Figure 10C). Western blot analysis showed an increase in total (Tau5), phosphorylated (CP13), and C-terminal truncated tau (TauC3) protein with Hcy treatment (Figure 10D). Dot blot analysis revealed that Hcy treatment increased TOC1 positive tau in cultured mouse neurons (Figure 10E). 

### 2.8. Induction of Hyperhomocysteinema in P301L Mice Increased Tau Phosphorylation, Cleavage and Oligomerization, an Effect Reversed by S-Adnosylmethionine Supplementation

**Figure 11:**
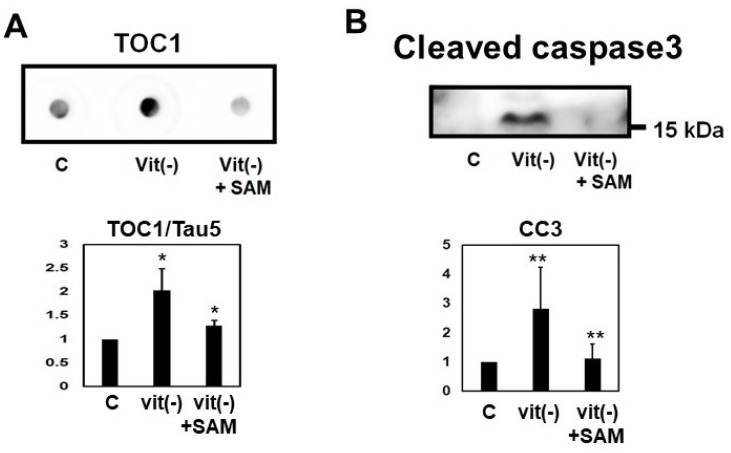
Induction of hyperhomocysteinema (HHCy) in P301L mice increased tau oligomerization, an effect reversed by S-adnosylmethionine (SAM) supplementation. The P301L mice fed with vitamin B_6_, B_12_, and folate-deficient chow (Hcy-diet), which is known to induce HHCy, exhibited increased of levels of oligomeric tau shown by dot blot analysist (**A**). Cleaved caspase 3 was also increased in HHCy mice (**B**). Oligomeric tau and cleaved caspase were reversed by the addition of SAM to the Hcy-diet (**A**,**B**). Results are presented as mean ± SD, ** *p* < 0.01, * *p* < 0.05. *N* = 5. C mice fed with control diet; Vit(−): mice fed with vitamin B_6_, B_12_, and folate-deficient diet; Vit(−) + SAM: mice fed with vitamin B_6_, B_12_, and folate-deficient diet with supplementation of SAM.

The P301L mice fed with vitamin B_6_, B_12_, and folate deficient chow (Hcy-diet), which is known to induce HHCy, exhibited increased levels of oligomeric tau which was shown by dot blot analysis (Figure 11A). Cleaved caspase 3 was also increased in HHCy mice (Figure 11B). Oligomeric tau and cleaved caspase 3 increases were reversed by the addition of SAM to the Hcy-diet (Figure 11A,B).

### 2.9. Tau Accumulation Induced by Homocysteine Was Reversed by the Addition of Folic Acid

Hcy disturbed the differentiation of cephalic neural crest cells into smooth muscle cells, and caused increased outgrowth and proliferation. However, folic acid (FA) supplementation with Hcy prevented this outgrowth and proliferation of cephalic neural crest cells, and these cells regained the ability to differentiate into smooth muscle cells [27]. We examined the effects of FA (90 µM) with 100 µM Hcy using tau expressing cells, and found that FA supplementation reversed the tau accumulation induced by Hcy treatment (Figure S2).

### 2.10. Hcy Treatment Inactivates the 20S Proteasome

**Figure 12:**
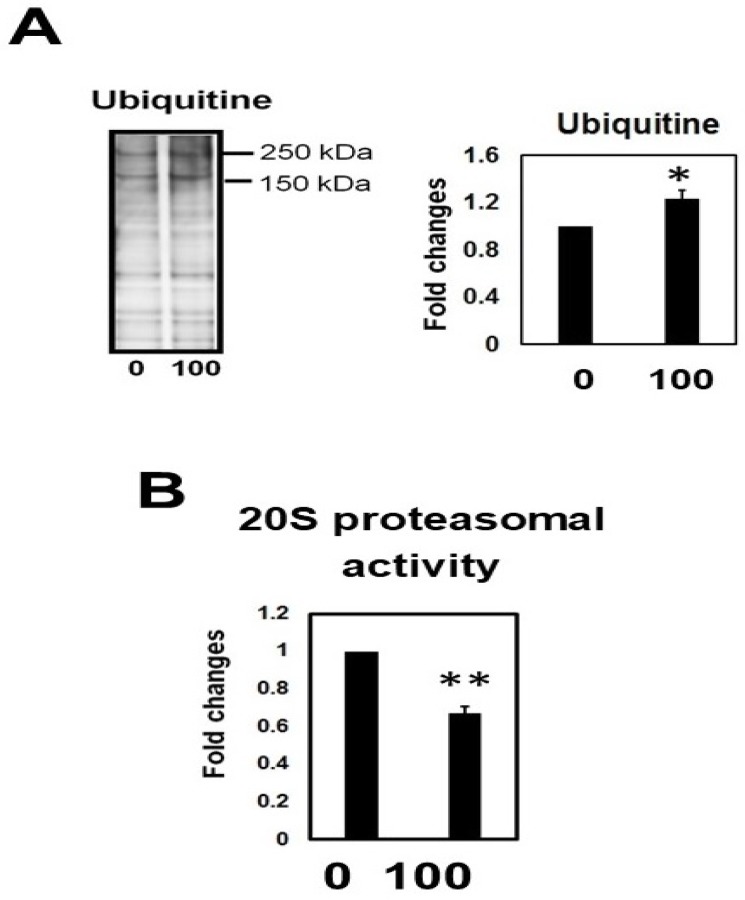
Homocysteine (Hcy) downregulated the ubiquitin proteasomal system (**A**). 0: 0 µM of Hcy, 100: 100 µM homocysteine. The amount of ubiquitinated protein is increased. *N* = 4, * *p* < 0.05, Bar: + SD (**A**). 20S proteasome activity (chymotrypsin-like activity) also was downregulated. *N* = 4, ** *p* < 0.01, Bar: ± SD (**B**).

The amount of ubiquitinated proteins was measured by immunoblotting lysates from cells treated with or without Hcy. Hcy significantly increased total ubiquitin levels in M1C cells (Figure 12A). Proteasome activity assays demonstrated a significant reduction of chymotrypsin-like activity after Hcy treatment (Figure 12B). These data suggest that Hcy causes a downregulation of proteasomal activity and increase of ubiquitinated proteins.

## 3. Discussion

Folate and vitamin B_12_ are cofactors in one carbone metabolism, during which they promote the remethylation of Hcy. Vitamin B_12_ and folate deficiency inhibits metabolism from Hcy to methionine and causes HHCy. Vitamin B_6_ deficiency also inhibits the conversion of Hcy to cystathionine and causes HHCy. Hcy exerts its intracellular effects through a number of pathways that can facilitate and/or directly cause neuronal dysfunction and death. For example, increased Hcy causes dysfunction of synapses and death of neurons by accelerating DNA breakdown and activating apoptotic signaling effectors, including caspases, p53, and Bax [28,29]. Hcy also induces release of cytochrome C, dysfunction of mitochondria, endoplasmic reticulum stress, and neurotoxicity by acting as an endogenous *N*-methyl-d-aspartate (NMDA) receptor activator [28,29,30,31]. Despite the widespread involvement of Hcy in these pathways, it remains relatively unclear exactly how Hcy leads to the formation of toxic tau species.

We found that Hcy increased several phosphoepitopes in tau, while simultaneously increasing an inactive form of PP2A. Previously, Li et al. reported that by diet-induced HHCy promoted tau protein phosphorylation at T231/S235 detected by the AT180 and AT270 antibodies, and no differences in total tau protein levels with or without HHCy in 3×Tg mice [15]. Zhang et al. reported that Hcy inactivated PP2A, and increased phosphorylation levels of tau protein (pS396) in increased plasma Hcy rat model by vena caudalis injection [16]. Chan et al. reported that folate deprivation, which causes HHCy, increased phosphorylated tau (PHF-1) and cytotoxic calcium influx, but they did not examine the direct effects of Hcy on kinase activities or tau aggregation [32]. We extend these data by showing that Hcy also activates GSK3β. The simultaneous activation of tau phosphokinase and inactivation of a protein phosphatase could enhance abnormal tau phosphorylation, which may affect its conformation and facilitate tau aggregation [18]. 

Here, we also demonstrate that excess Hcy induces caspase activation, as well as both caspase-mediated tau cleavage and tau oligomers formation, which are associated with tau toxicity. The C-terminus of tau prevents tau aggregation in vitro and C-terminal cleavage by caspase can stimulate polymerization of tau [17,33]. Importantly, caspase-cleaved tau species are associated with cell toxicity and enhanced tau secretion [25,34]. The Hcy-mediated increase in caspase-cleaved tau (at D421) was associated with increased tau in the sarkosyl insoluble fraction. 

In our study, Hcy produced an increase in total tau protein, which suggests that high Hcy levels impairs tau turnover. The ubiquitin proteasome system is an important cellular mechanism that controls the levels of intracellular protein and removes mutant, damaged, and misfolded proteins to control the quality of protein both in the nucleus and cytoplasm. In postmortem AD brains, the 20S proteasome (core particle) and trypsin-like activity was remarkably decreased [35]. We found that Hcy caused an increase in ubiquitinated protein and a downregulation of 20S proteasome activity. Indeed, it was reported that aggregated tau may interact with and inhibit the 20S proteasome [36]. These results imply that perturbation of the ubiquitin proteasome system by Hcy may contribute, at least in part, to the accumulation of abnormally modified and oligomeric tau. 

Our results also showed that Hcy contributed to increased high molecular weight tau (>120 kDa) in the sarkosyl soluble fraction. Dot blot analysis and immunocytochemical study demonstrated that TOC1 positive oligomeric tau was increased by Hcy. TOC1 is a conformational dependent antibody whose epitope spans amino acid residues 209–224 of full length 4R2N tau [37]. 

Mice fed the Hcy diet also show an increase in TOC1 positive oligomeric tau shown by dot blot analysis (Figure 11). Oligomeric tau is considered a key player in AD progression [38,39,40,41], and tau oligomers appear in the early phase of neuronal cell pathology in several tauopathies, including AD [3,41,42,43]. Oligomeric tau species induce synaptic and mitochondrial dysfunction [40,41], and axonal transport impairments [44], and have cytotoxic effects on neurons [38,39,40]. Injection of tau oligomers in the brain facilitated aggregation of endogenous tau, resulting in the propagation of tau pathology [45]. In our study, Hcy-induced cell toxicity led to an elevation in TOC1 positive oligomeric tau in M1C cells and neurons. 

Increased tau oligomerization and caspase activation in the P301L mice fed with Hcy-diet were reversed by the addition of SAM to the Hcy-diet in our study. In cell culture, FA addition reversed Hcy induced tau accumulation. Previously, it was reported that anti-homocysteic acid antibody therapy in Vitamin B_6_ deficient mice improved cognition [46].

Recent evidence suggests that elevated Hcy causes a number of conditions that may lead to neurodegenerative disorders, including AD [11] and Parkinson’s disease (PD) [47]. HHCy can also be the cause of gray matter atrophy in humans [48]. HHCy is also a risk factor of stroke [49,50], and myocardial infarction [51]. Actually, HHCy is associated with endothelial dysfunction [52], impaired nitric oxide activity [53], increased oxidative stress [54], and cerebral microangiopathy [55]. However, not all of the disorders for which HHCy is a risk factor are accompanied by tauopathy. Thus, further studies are needed to better understand why HHCy is associated with tauopathy in some populations but not in others. 

**Figure 13:**
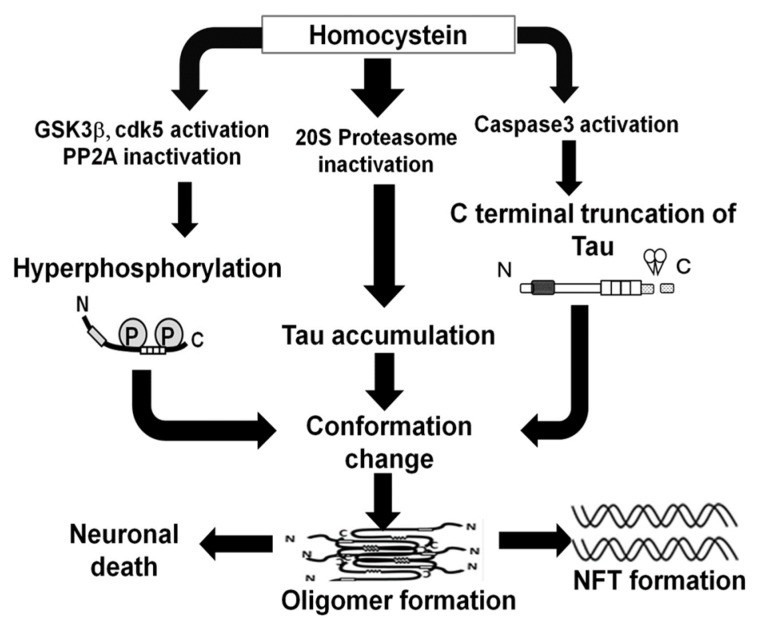
Schematic of expected effects of tau aggregation induced by homocysteine (Hcy). Hcy decreased 20S proteasome activity and tau accumulation in the cell. Hcy increased phosphorylation levels of tau protein via GSK3β and cdk5 activation or PP2A inactivation. Hcy activates caspase 3 and increased C-terminal truncated tau by caspase. Increased C-terminal truncated tau and phosphorylated tau may accelerate conformational changes in tau, and accelerate tau oligomerization. Oligomeric tau can be cause of neuronal death, or forms neurofibrillary tangle (NFT).

Our results suggested that HHCy, which can be induced by folate, vitaminB_6_, or B_12_ deficiency, causes misregulation of kinases and phosphatases that affect tau, caspase activation, and impaired proteasomal function. Accordingly, tau accumulation occurs, phosphorylation of tau increases, caspase-cleaved tau appears, and tau oligomers form, all of which are associated with cell toxicity (Figure 13). Collectively, our findings suggest that modulation of Hcy levels is a potentially viable mechanism to mitigate the deleterious effects of abnormal toxic tau species in tauopathies. 

## 4. Materials and Methods 

### 4.1. Materials 

Lab-Tek II chambered cover glass was from Thermo Fisher Scientific (Rochester, NY, USA). Unless indicated, tissue culture ware was purchased from BD Biosciences (Franklin Lakes, NJ, USA). L-Hcy (SE-280883A) was from Santa Cruz Biotechnology (Dallas, TX, USA). G418 was from Life Technologies (Gaithersburg, MD, USA). Neurobasal medium was from Life Technologies (Grand Island, NY, USA). Protease inhibitor cocktail was from Roche (Mannheim, Germany). Enhanced Chemiluminescence Prime (ECL Prime) was from Amersham (Buckingham shire, UK). Cell Titer-Glo Luminescent Cell Viability Assay Kit was from Promega (Madison, WZ, USA). Suc-LLVY-AMC fluorogenic substrate was from Biomol International (Plymouth Meeting, PA, USA). StepOnePlus Realtime PCR System was from Applied Biosystems (Foster City, CA, USA). Other chemicals were procured from Sigma when not mentioned specifically. 

### 4.2. Antibodies

**Figure 14:**
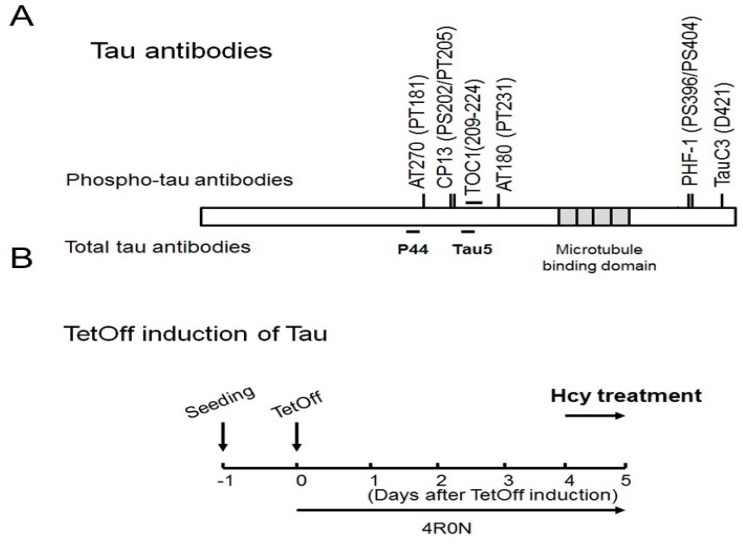
Tau antibodies used in this study and the experimental timeline. Schematic depiction of human 4R0N tau and different epitopes recognized by tau antibodies used in this study (**A**). Schedule of induction of tau expression in M1C cells with tau controlled under a tetracycline off (TetOff) expression system. On Day 0, induction of tau expression was started by reducing the concentration of tetracycline in the medium from 2000 to 1 ng/mL. On Day 4, L-Hcy treatment was started and on day 5 the cells were harvested (**B**).

Several well-characterized tau antibodies were used in these studies and the epitopes are shown in Figure 14A [6,10,24,56,57,58,59]. Phosphorylated tau specific monoclonal antibodies, PHF-1, and CP13, were generous gifts from Peter Davies (The Feinstein Institute for Medical Research, Manhasset, NY, USA). Tau5 [60], TauC3 [24], and Tau Oligomeric Complex 1 antibody (TOC1, recognizes non-filamentous tau multimers) [42,61] were developed by Lester Binder at Northwestern University. Rabbit anti-tau phosphoserine 199/202 (PS199/202) polyclonal antibody was from EMD Millipore (catalog #AB9674, Temecular, CA, USA). Monoclonal antibodies for glyceraldehyde 3-phosphate dehydrogenase (GAPDH) was from Millipore (catalog #MAB374, Billerica, MA, USA), PP2A and demethylated PP2A (DPP2A) were form Santa Cruz Biotechnology (catalog #sc-80665, and #sc-13601), and ubiquitin was from Cell Signaling (catalog #3936, Danvers, MA, USA). Polyclonal antibodies for GSK3β, phospho-GSK3β (Ser9) (p-GSK3β), and cleaved caspase 3 were from Cell Signaling (catalog #9315, #9336, and #9661). These antibodies were used at the following dilutions: Tau5 (1:1000), PHF-1 (1:200), CP13 (1:200), AT180 (1: 1000), AT270 (1:1000), TauC3 (1:2000), TOC1 (1:1000), anti-GAPDH (1:2000), anti-GSK3β (1:1000), anti-pGSK3β (1:500), anti-PP2A (1:1000), anti-DPP2A (1:1000), anti-cleaved caspase 3 (1:500), and anti-ubiquitin (1:1000). 

### 4.3. Cell Culture

We plated M1C cells (originated from BE-(2)-M17D cells; human neuroblastoma) that express 4R0N tau via a tetracycline off (TetOff) inducible expression system [6,10,56,57,62] at 2.6−3.5 × 10^4^ cells/cm^2^ in each culture dish in dimethyl sulfoxide (DMEM) with 10% fetal bovine serum, G418 (400 µg/mL), and tetracycline (Tet) (2 µg/mL). Tau expression was started 24 h after plating by decreasing the concentration of Tet to 1 ng/mL (TetOff induction). L-Hcy was diluted in water and added to M1C cells on 3–4 days after TetOff induction. Cells were collected at 5 days after induction (Figure 14B). 

### 4.4. Animals and Treatment

All animal procedures were approved by the National Center for Geriatrics and Gerontology (NCGG), and University of Fukui. We utilized the well-characterized rTG4510 mouse line as a model of tauopathy (Jackson Lab, Bar Harbor, ME, USA) [63]. Tg4510 mice have the tau responder and activator transgenes that drive overexpression of the human four-repeat tau gene, 4R0N, containing the P301L mutation, which causes an inherited form of tauopathy in humans [1,63]. Mice used were mixed genders of progeny crosses between activator transgene in a 129 background strain and the tau responder transgene in an Friends virus B (FVB) background. Mice were provided food/water ad libitum, and kept on a light/dark cycle (12 h/12 h) at 23 °C. All mice were purchased from Charles River Laboratories Japan, Inc. (Yokohama, Japan). The mice were randomized into 3 groups. The control group mice (*N* = 5) were given standard rodent chow, whereas the Hcy-diet group mice (*N* = 5) were given a standard rodent chow deficient in folate (<0.2 mg/kg), vitamin B_6_ (<0.1 mg/kg), and B_12_ (<0.001 mg/kg), which is known to induce HHCy in mice [64]. The diet was prepared by a commercial vendor (Research Diet) and matched for calories. The third group received the Hcy-diet and SAM supplement at 80 mg/kg mouse/day in the water. Starting at 3 months of age, mice received the diet for 4 weeks until they were 4 months old. During this study, mice in all groups gained weight regularly, and no significant differences in weight were detected in the 3 groups. After sacrifice, the brains were removed and dissected in two sample sets per hemisphere. Sample one contained the cerebral cortex and hippocampus, and sample two contained the basal ganglia, hypothalamus, brainstem, and cerebellum. The brain samples were used for the fractionation studies described below. (The Committee for Animal Research of the National Center for Geriatrics and Gerontology Approval code:29-2; Date: 9 March 2017)

### 4.5. Fractionation Analysis

Harvested cells were homogenized in Tris buffer with protease and phosphatase inhibitors (25 mM Tris HCl, pH 7.4, 50 mM sodium pyrophosphate, 30 µM β-glycerophosphate, 30 µM sodium fluoride, and protease inhibitor cocktail), and 1 µM ethylenediamine tetra acetic acid (EDTA), and centrifuged at 180× *g* for 15 min to remove nuclei and debris. The supernatants were then fractionated according to solubility in Tris buffer or 1% sarkosyl to obtain SN1, SN2, and S/P fractions, as described before [6,10,57]. Supernatants were obtained by centrifugation of lysates at 150,000× *g* for 15 min at 4 °C and collected as SN1 fractions. The pellets were homogenized in buffer with 0.8 M NaCl, 10% sucrose, 10 mM Tris/HCl pH 7.4, 1 mM ethylene glycol tetraacetic acid (EGTA), protease inhibitor cocktail, and 1% sarkosyl. After the centrifugation as above, supernatants (SN2) were collected, and the pellets (S/P: sarkosyl insoluble fraction) were resuspended in Tris buffer and immediately used for Western blot analysis or stored at −20 °C. 

### 4.6. Western Blotting and Dot Blot 

Preparation of samples was done in Laemmli sample buffer with or without 1% β-mercaptoethanol (βME). Concentrations of protein in samples were measured using the bicinchoninic acid assay (Thermo Scientific, Rockford, IL, USA). Equivalent amount of protein (10 µg) was loaded (equivalent to 0.1–0.2 × 10^5^ cells), separated by SDS-PAGE on 10% gels, and transferred onto polyvinylidene difluoride (PVDF) membranes (Immobilon P; Millipore). Dot blot samples were directly spotted onto PVDF membranes at 2 µg of protein [61]. Both Western and dot blots were incubated in blocking solution. After blocking, blots were incubated with several antibodies, including tau antibodies for 1 h at room temperature (RT). Then, the blots were washed by Tris buffer saline with 0.1% tween 20 three times. Then, the blots were incubated with horseradish peroxidase-conjugated sheep anti-mouse IgG, goat anti-rabbit IgG, or goat anti-mouse IgM for the TOC1 antibody at RT 30 min. After the last wash, immunoreactivity was developed with enhanced chemiluminescence (ECL) Prime reagent [6,10], and imaged using a digital imaging system. ImageJ software (version 1.51, NIH, Bethesda, MD, USA) was used for making densitometry measurements of immunoreactive bands. Data were normalized with GAPDH and total tau protein as indicated.

### 4.7. Immunocytochemistry

After the TetOff induction, cells grown on Lab-Tek chambered cover glass were treated with L-Hcy (100 µM) for the last 24 h of the TetOff induction period. Then, cells were fixed with 4% paraformaldehyde/0.1 M phosphate buffer (PB). We rinsed the cells with PBS, and permeabilization was performed using 0.25% Triton X-100/PBS. Next, we blocked the cells in blocking solution (3% goat serum and 1% bovine serum albumin in PBS) and incubated in P44, CP13, PS199/202, TauC3, or TOC1 antibody solution, followed by Alexa 488 anti-mouse IgG and Alexa 594 anti-rabbit IgG, or Alexa 488 goat-mouse IgM in blocking solution (Molecular Probes, Eugene, OR, USA). A confocal laser microscope (version 09-2004, TCSSPII; Leica, Heidelberg, Germany) was used to image the cells. The number of cells positive for each tau antibody was calculated in 4 randomly chosen visual fields per well and each experimental group was repeated with 4 wells. 

### 4.8. Morphological Study and Cell Viability Assay

Cultured M1C cells grown in 24-well plates at the density of 2.6–3.5 cells × 10^3^/cm^2^ were subjected to morphological studies with or without L-Hcy treatment after the TetOff induction. Phase contrast images of M1C cells were obtained using an inverted microscope (IX-70: Olympus, Tokyo, Japan) equipped with a digital camera (DP-70: Olympus), and the images were processed using Adobe Photoshop Elements Version 13 (Adobe, San Jose, CA, USA) for publication. Cell body area was measured in each visual field (3 randomly chosen visual fields were assessed per well and each experimental group was repeated with 3 wells) by outlining the entire area of the somata (for area) using ImageJ software. 

The Cell Titer-Glo assay (Promega, Cat#G7570), which utilizes ATP to assay cell viability, was used to measure the extent of cell toxicity according to the manufacturer’s instructions. Briefly, M1C cells with TetOff induction or mouse neurons were cultured in 384 well plates with or without Hcy. Twenty-five µL of mixed reagents was added to each well, and luminescence was measured with a SpectraMax M5 microplate reader (Molecular Devices, San Jose, CA, USA). Each experimental group was repeated with 4 wells. 

### 4.9. mRNA Expression

Quantitative real time PCR (qPCR) was done according to our previous report and all samples were run three times [6,10]. Briefly, 20 ng of cDNA per sample was used in a 20 μL reagent mix, including previously described primers [10], with the Applied Biosystem StepOnePlus Realtime PCR system (#4370048). Changes in gene expression levels were obtained by the ∆Ct method described before [10,65]. 

### 4.10. Primary Neuronal Cell Culture

Primary neuron culture was performed according to previous reports [6,10,21]. Briefly, cortical neurons from Slc:ICR mice, which were male and female, were isolated on embryonic day 16 and plated on Lab-Tek II 8 well chambered coverglass at a density of 5 × 10^4^ cells/well for immunocytochemistry or in 3.5-cm plastic dishes at a density of 4 × 10^5^ cells/dish for western blotting. Coverglasses and plastic dishes were precoated with 0.2% polyethyleneimine (Sigma, St. Louis, MO, USA). Primary neurons were cultured in serum-free Neurobasal medium and exposed to 5 µM Ara C to reduce non-neuronal cell proliferation [6,10,21]. All experiments were performed using cultures highly enriched in neuronal cells (>95% neuronal), as confirmed with anti-NeuN (Millipore) immunocytochemistry.

### 4.11. 20S Proteasome Activity Assay 

We incubated lysates (50 µg total protein/sample) at 37 °C for 60 min in the dark with the proteasome activity assay reaction buffer (50 µL/sample; 50 mM 2-[4-(2-Hydroxyethyl)-1-piperazinyl]ethanesulfonic Acid (HEPES) (pH 7.5), 5 mM MgCl2, 150 mM NaCl, 20% glycerol, 5 mM sodium pyrophosphate, 30 mM β-glycerophosphate, 30 mM sodium fluoride, and a protease inhibitor cocktail) containing 100 µM Suc-LLVY-AMC fluorogenic substrate (Enzo Life Sciences, Inc., Farmingdale, NY, USA) in 96 well plates for measuring chymotrypsin-like activity [62,66]. Fluorescence signals were measured using a 380 nm excitation filter and 460 nm emission filter to assess chymotrypsin-like activity using a SpectraMax M5 microplate reader. 

### 4.12. Statistical Analysis

Data are shown as the mean ± standard deviation (SD) from the minimum of three experiments. Statistical comparisons were assessed by two-tailed Student’s *t*-test (IBM SPSS Statistics, version 22, IBM Corp., Armonk, NY, USA) or by a two-tailed one-way analysis of variance (ANOVA) with the Student–Newman–Keuls post-hoc test. Differences were considered significant when *p* < 0.05 (* *p* < 0.05, ** *p* < 0.01, n.s.: not significant). 

## 5. Conclusions

HHCy, which can be induced by folate, vitamin B_6_, or B_12_ deficiency, causes misregulation of kinases and phosphatases that affect tau, caspase activation, and impaired proteasomal function. Accordingly, tau accumulation occurs, phosphorylation of tau increases, caspase-cleaved tau appears, and tau oligomers form, all of which are associated with cell toxicity.

## Supplementary Materials

Supplementary materials can be found at http://www.mdpi.com/1422-0067/19/3/891/s1.

## Abbreviations

AD	Alzheimer’s disease
HHCy	Hyperhomocysteinemia
Hcy	Homocysteine
SAH	S-adenosylhomocysteine
SAM	S-adenosylmethionine
cdk5	cyclin-dependent kinase 5
PP2A	protein phosphatase 2A
GSK3β	glycogen synthase kinase 3β
