# Supplementary material for: Homocysteine Increases Tau Phosphorylation, Truncation and Oligomerization
Source: Int J Mol Sci. 2018 Mar 17;19(3):891. doi: 10.3390/ijms19030891 (PMC5877752; doi:10.3390/ijms19030891)
Supplement: Supplementary file 1 [file ijms-19-00891-s001.pdf]

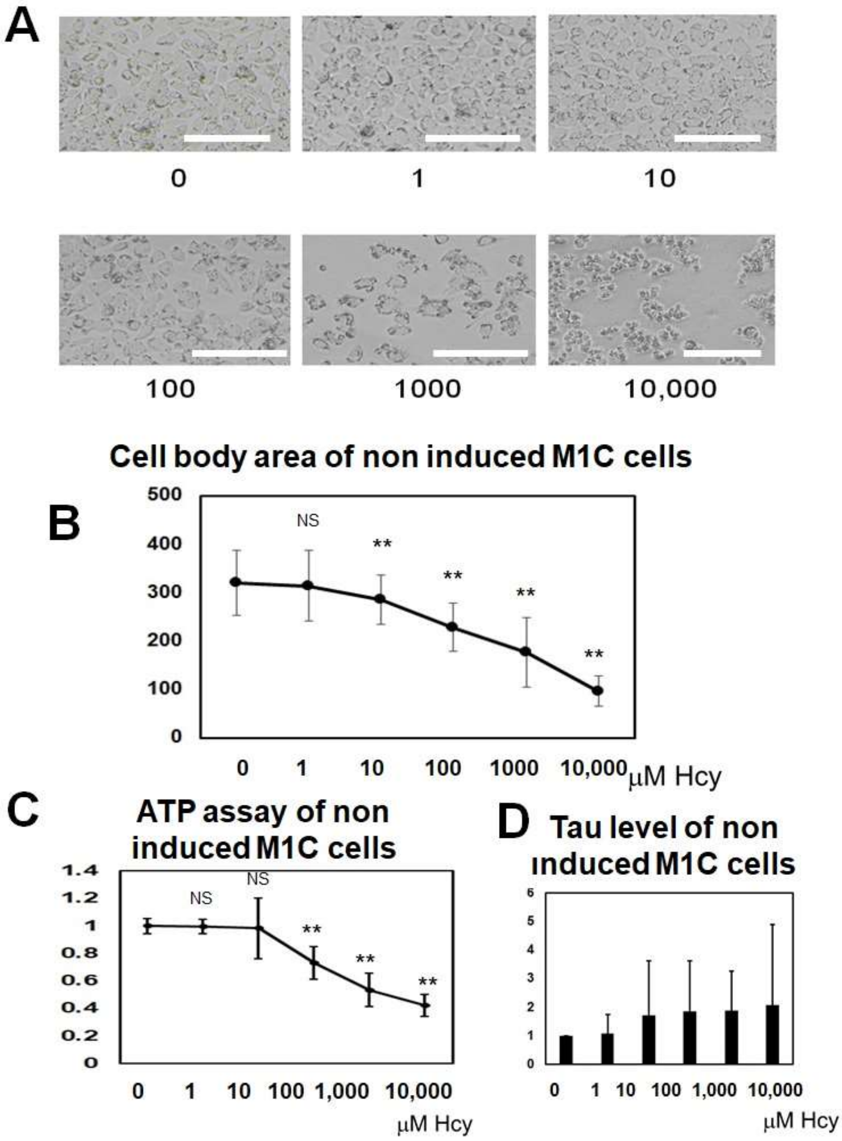

Figure S1. Homocysteine (Hcy) has cytotoxic effects on M1C cells without tau induction (i.e., maintained on 2 μg/mL Tet). M1C cells were exposed to 1, 10, 100, 1000, and 10,000 μM L-Hcy for 24 h. Then, M1C cells treated with or without L-Hcy were subjected to morphological studies. Qualitatively, 100–10,000 μM L-Hcy induced shrinking of the cell body. Bar 100 μm (**A**). Quantitative measurements of cell body area showed that 10–10,000 μM Hcy decreased cell area in dose-dependent fashion. Results are presented as mean ± SD, ** $p < 0.01$ (**B**). The ATP assay (**C**) showed that 100 to 10,000 μM Hcy caused a reduction of cell viability. Results are presented as mean ± SD, ** $p < 0.01$ (**C**). Total tau levels in non-induced M1C cells by 1–10,000 μM of Hcy treatment were quantitated. Results are presented as mean ± SD (**D**).

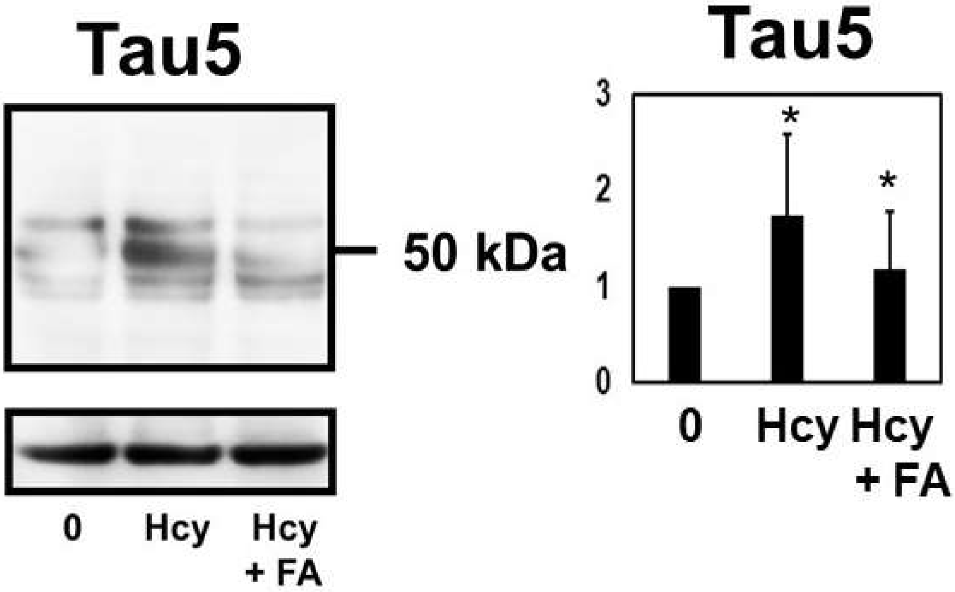

Figure S2. Homocysteine (Hcy) induced tau accumulation was reversed by the addition of folic acid (FA). We examined the effects of FA (90 μM) with 100 μM Hcy using tau expressing cells. FA supplementation reversed the tau accumulation induced by Hcy treatment. Results are presented as mean ± SD, * P <0.05. N = 5. NI: non induced cells, Hcy: 100 μM Hcy, Hcy + FA: 100 μM of Hcy + 90 μM of FA.
